# On the Cryptanalysis of a Latin Cubes-Based Image Cryptosystem

**DOI:** 10.3390/e23020202

**Published:** 2021-02-07

**Authors:** Rong Huang, Hao Liu, Xiaojuan Liao, Aihua Dong

**Affiliations:** 1 College of Information Science and Technology, Donghua University, Shanghai 201620, China; dongaihua@dhu.edu.cn; 2 Engineering Research Center of Digitized Textile & Apparel Technology, Ministry of Education, Donghua University, Shanghai 201620, China; 3 College of Information Science and Technology, Chengdu University of Technology, Chengdu 610059, China; liaoxiaojuan18@cdut.edu.cn

**Keywords:** cryptanalysis, chosen-plaintext attack, image cryptosystem, Latin cubes

## Abstract

Based on orthogonal Latin cubes, an image cryptosystem with confusion–diffusion–confusion cipher architecture has been proposed recently (*Inf. Sci.* **2019**, *478*, 1–14). However, we find that there are four fatal vulnerabilities in this image cryptosystem, which leave open doors for cryptanalysis. In this paper, we propose a reference-validation inference algorithm and design screening-based rules to efficiently break the image cryptosystem. Compared with an existing cryptanalysis algorithm, the proposed method requires fewer pairs of chosen plain-cipher images, and behaves stably since different keys, positions of chosen bits and contents of plain images will not affect the cryptanalysis performance. Experimental results show that our cryptanalysis algorithm only requires $\;\sqrt[{3}]{8\times H\times W}+3\;$pairs of chosen plain-cipher images, where H×W represents the image’s resolution. Comparative studies demonstrate effectiveness and superiority of the proposed cryptanalysis algorithm.

## 1. Introduction

One image is worth more than ten thousand words. How to protect image contents from illegal accesses has become a crucial security issue for the practical applications such as virtual meeting, video surveillance or telemedicine, especially when we have entered the era of big data. Cryptography is a cornerstone in the field of information security. Conventional data encryption techniques, e.g., DES (data encryption standard), AES (advanced encryption standard) and IDEA (international data encryption algorithm), are inappropriate for image encryption applications because there are high redundancies and strong correlation among adjacent pixels [1,2]. Permutation-and-diffusion cipher architecture, which alternately shuffles pixel positions and changes pixel values, has the capability to reduce the redundancy and the correlation, thereby playing a central role in many image encryption algorithms [3,4,5,6]. 

Naturally, an image can be encoded as a three-dimensional (3D) bit matrix, in which bits are the smallest elements representing digital information. Some methods [7,8,9,10,11,12,13,14] have extended the permutation-and-diffusion cipher architecture to 3D version, in which the bit-level permutation not only shuffles the bit positions but also changes the pixel values at the same time. Zhu et al. [7] employed Arnold cat map for bit-level permutation and Logistic map for diffusion. Zhang et al. [8] invented a collision-free random bidirectional visiting mechanism by coupling Chen chaotic system with Arnold cat map, and developed a new hybrid 3D permutation rule. In the image cryptosystem [9], multiple chaotic maps were used to control the bit-level row/column-wise permutation. Cai et al. [10] proposed a plaintext-related random-access mechanism that scrambles the 3D bit matrix based on a mixture of three chaotic maps. Gan et al. [11] obtained a random mapping sequence from Chen chaotic system, and designed a tailor-made multilevel quantizer for generating diffusion matrices. Pak et al. [12] improved the original Logistic map and Sine map by introducing a differential amplification operation, and then built a linear–nonlinear–linear conversion structure geared towards a bit-level image cryptosystem. Xu and Tian [13] constructed a confusion-diffusion-confusion cipher architecture, and grouped three orthogonal Latin cubes together to form a 3D random permutation table. Shahna and Mohamed [14] performed the image permutation at the pixel and bit levels, which combines a key-governed scan pattern with a cyclic shift operation. 

Cryptanalysis, which is the opposite of cryptography, focuses on exploring and exposing vulnerabilities in a cryptosystem. Indeed, some of the above works [7,8,9,10,11,12,13] have been found to be insecure. Zhang and Wang [15] developed a differential attack that can effectively bypass Zhu’s Arnold cat map [7]. Then, they put forward an improved proposal, in which flipping operations are embedded into the permutation phase, and the connection between plain pixels and keys is established by swapping the order of the permutation and diffusion phases. Later, Wang and Zhao [16] found that Zhang’s improved proposal [15] is still insecure. A reverse differential attack can exactly counteract the order exchange between the permutation and diffusion phases so as to equivalently reconstruct the random mapping sequence. Wu et al. [17] pointed out that the hybrid 3D permutation of Zhang’s image cryptosystem [8] fails to move the bit at the origin, and the diffusion phase merely depends on a weak CBC (cipher block chaining) mode. To break Zhang’s image cryptosystem [8], Li et al. [18] first separated the permutation and diffusion phases by designing a special plain image, and then revealed functionally equivalent keys by using the chosen-plaintext attack. In the same way, Li et al. [19] broke Pak’s work [12] by comparing the differences between several pairs of chosen plain-cipher images. After analyzing the image cryptosystem [9], Liu et al. [20] reported that the correlation information among adjacent plain pixels cannot be erased by the row/column-wise permutation, thereby leaving an open door for the known-plaintext attack. Wen and Yu [21] ascertained that, in the image cryptosystem [9], keys are not associated with the plain images. Exploiting this security flaw, they launched a cracking attack in a divide-and-conquer manner. Recently, Zhang and Yu [22] demonstrated that the process of generating the orthogonal Latin cubes in [13] is independent of the plain images. This allows an adversary to expose the random mapping sequence by using the chosen-plaintext attack. 

In this paper, we reinvestigate the security loopholes of Xu’s image cryptosystem [13]. Inspired by the chosen-plaintext attack, we propose an efficient reference-validation inference algorithm and design a screening-based rule to break Xu’s image cryptosystem [13]. Compared with Zhang’s work [22], our cryptanalysis algorithm requires fewer pairs of chosen plain-cipher images, and behaves stably since different keys, positions of chosen bits and contents of plain images will not affect the cryptanalysis performance. Experimental results demonstrate the effectiveness and the superiority of the proposed cryptanalysis algorithm. 

The rest of this paper is organized as follows. Section 2 gives a brief introduction to Xu’s image cryptosystem [13], which is the attack target in this study. In Section 3, we first summarize the existing vulnerabilities of [13], and then describe the proposed cryptanalysis algorithm in detail. In Section 4, we conduct extensive experiments and exhibit corresponding results. Last section concludes this paper. 

## 2. Review of Target Image Cryptosystem 

In this section, we briefly introduce the TIC (target image cryptosystem) [13]. Overall, it consists of three encryption phases based on Latin cubes, namely pre-permutation, diffusion, and post-permutation. 

For better readability, we use bold uppercase symbols (e.g., **P**) and bold lowercase symbols (e.g., **p**) to represent cubes and sequences, respectively. Let *P*(*x, y, z*) denote the element of **P** at the coordinate (*x*, *y*, *z*). Let *p*(*n*) denote the *n*th element of **p**. Non-bold italic font (e.g., *n* and *N*) denotes scalars, and Greek letters (e.g., *μ*) stand for the keys of an image cryptosystem. Bold calligraphic capital letters (e.g., N) is used to represent sets. Let ℤ represent the ring of integers.

Consider an 8-bit plain image with *H* × *W* resolution, where the total number of bits is 8 × *H* × *W*. Let *N* denote the side length of a bit-cube, whose value equals$\;\sqrt[{3}]{8\times H\times W}$. For simplicity, Xu and Tian [13] assume that the image size *H* and *W* are appropriate values to ensure that the side length *N* is an integer. Reshape the plain image into the plain bit-cube, denoted by **P**. 

In the TIC [13], Logistic map is adopted to generate a chaotic sequence, denoted by **r** = [*r*(0), *r*(1), …, *r*(*N −* 1)]. The definition of the Logistic map can be expressed as

**Figure 1:**
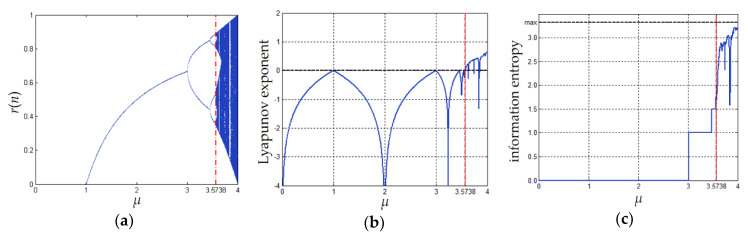
Chaotic behaviors of the Logistic map: (**a**) bifurcation diagram; (**b**) diagram of Lyapunov exponent; (**c**) diagram of information entropy. Note that when calculating the information entropy, we simply discretize *r*(*n*) through the formula ⎣r(n)·10⎦.

*r*(*n +* 1) = *μ·r*(*n*) (1 − *r*(*n*)), (1)

where *n* = 0, 1, …, *N* *−* 1, and *κ* = *r*(−1) is the initial condition. In Equation (1), *μ* is the system parameter. When its value lies in the interval (3.573815, 4], the system exhibits chaotic properties, including ergodicity and high sensitivity to the initial conditions [13]. Figure 1 shows diagrams of bifurcation, Lyapunov exponent, and information entropy of the Logistic map. More details can be found in [23].

The chaotic sequence **r** is first sorted in ascending order, and then the sorted result is used to form a random mapping sequence, denoted by **s** = [*s*(0), *s*(1), …, *s*(*N −* 1)]. Specifically, **s** represents the random mapping relations between **r** and its sorted counterpart. Clearly, *s*(*n*) is an integer lying in the interval [0, *N −* 1]. By using the random mapping sequence **s**, three orthogonal Latin cubes, denoted by **L**_1_, **L**_2_ and **L**_3_, are generated as follows

(2) $$\{\begin{array}{l} L_{1}(x,\;y,\;z)=\alpha^{2}\times s(x)+\alpha \times s(y)+s(z),\\ L_{2}(x,\;y,\;z)=\beta^{2}\times s(x)+\beta \times s(y)+s(z),\;\\ L_{3}(x,\;y,\;z)=\gamma^{2}\times s(x)+\gamma \times s(y)+s(z), \end{array}$$

where *L*_1_(*x*, *y*, *z*), *L*_2_(*x*, *y*, *z*) and *L*_3_(*x*, *y*, *z*) are the elements of **L**_1_, **L**_2_ and **L**_3_ at the coordinate (*x*, *y*, *z*), respectively. In Equation (2), the addition operator “+” and the multiplication operator “×” are both defined on the ring ℤ/Nℤ. Note that, in the TIC [13], *N* is fixed to 128 so that the addition and multiplication operators are originally defined on the finite field GF(2^7^). However, it is too strict to consider a constant side length. In a practical image cryptosystem, *N* may not be a power of a prime number. Therefore, in this paper, we define the two operators on the ring ℤ/Nℤ rather than on a finite field. 

The three control parameters, namely *α*, *β*, and *γ*, in Equation (2), must be different nonzero numbers within the ring ℤ/Nℤ. This is a necessary and sufficient condition for the three Latin cubes to be mutually orthogonal. The orthogonality property means that each triple (*L*_1_(*x*, *y*, *z*), *L*_2_(*x*, *y*, *z*), *L*_3_(*x*, *y*, *z*)) appears only once after traversing all possible ones, where *x*, *y*, and *z* take values from [0, *N −* 1]. The orthogonality property ensures that all the mapping relations between (*x*, *y*, *z*) and (*L*_1_(*x*, *y*, *z*), *L*_2_(*x*, *y*, *z*), *L*_3_(*x*, *y*, *z*)) are one-to-one correspondences without collision, so that the orthogonal Latin cubes can be directly used for permuting a bit-cube. It is worth clarifying here that the initial condition *κ*, the system parameter *μ*, and the three control parameters *α*, *β*, *γ* collectively serve as keys in the TIC [13].

The first pre-permutation phase shuffles the bit positions of **P** based on the three orthogonal Latin cubes. Formally, the pre-permutation phase can be expressed as

*U*(*x*, *y*, *z*) = *P*(*L*_1_(*x*, *y*, *z*), *L*_2_(*x*, *y*, *z*), *L*_3_(*x*, *y*, *z*)), (3)

where U=[U(x, y, z)|x, y, z=0, 1, ⋯ , N−1] represents the permuted version of **P**.

The diffusion phase, which is executed after the pre-permutation phase, aims to flip a part of bits in **U** under the control of a random bit sequence. In the TIC [13], the random bit sequence is extracted from **L**_1_ through binarization

(4) $$B\left(x,\;y,\;z\right)=\{\begin{array}{ll} 0, & \mathrm{if}\;L_{1}(x,\;y,\;z)\;\geq N/2,\\ \;1, & \mathrm{otherwise}.\; \end{array}$$

In Equation (4), N/2 is the threshold. The binarized bit-cube is denoted by B=[B(x, y, z)|x, y, z=0, 1, ⋯ , N−1]. Reshape **U** and **B** into two one-dimensional (1D) bit sequences according to the same scanning order. Here, the 3D-to-1D coordinate transformation can be explicitly formulated as

*n* = *x* × *N*^2^+ *y* × *N* + *z.* (5)

The two resulting 1D bit sequences are denoted by **u** = [*u*(0), ⋯, *u*(*n*), ⋯, *u*(*N −* 1)] and **b** = [*b*(0), ⋯, *b*(*n*), ⋯, *b*(*N −* 1)], respectively. The diffusion phase is described as

*v*(*n*) = *u*(*n*) ⨁ *v*(*n −* 1) ⨁ *b*(*n*), (6)

where **v** = [*v*(0), ⋯, *v*(*n*), ⋯, *v*(*N −* 1)] is the diffused bit sequence, and *v*(−1) is initialized to 0. In Equation (6), the sign “⨁” represents bit-wise exclusive or (XOR) operator.

In the last post-permutation phase, a CPV (cipher-parity value), denoted by *t*, is first defined by

*t* = *v*(0) ⨁ *v*(1) ⨁⋯⨁ *v*(*N −* 1). (7)

Then, reshape the 1D diffused bit sequence **v** into a diffused bit-cube **V**. Here, the 1D-to-3D coordinate transformation for each bit position can be explicitly expressed as

(8) $$\{\begin{array}{l} x=⎣n/N^{2}⎦,\\ y=⎣n/N⎦\%N,\\ z=n\%N,\; \end{array}$$

where the floor sign “⎣·⎦” rounds down to the nearest integer of the number enclosed within the sign, while “%” represents a remainder operator. The three Latin cubes are reused in the following form to permute the diffused bit-cube **V**

(9) $$\{\begin{array}{ll} C(x,\;y,\;z)=V(L_{2}(x,\;y,\;z),\;L_{3}(x,\;y,\;z),\;L_{1}(x,\;y,\;z)),\; & \mathrm{if}\;t=0,\\ C(x,\;y,\;z)=V(L_{3}(x,\;y,\;z),\;L_{1}(x,\;y,\;z),\;L_{2}(x,\;y,\;z)),\; & \mathrm{otherwise}. \end{array}$$

In Equation (9), C=[C(x, y, z)|x, y, z=0, 1,…, N−1] represents the resulting bit-cube of the post-permutation phase. Finally, reshape **C** into an 8-bit cipher image with *H* × *W* resolution.

Decryption is composed of the inverse encryption operations, which are organized in a reverse order. 

## 3. Cryptanalysis

In this section, we first summarize the existing vulnerabilities of the TIC [13] from four aspects. Then, inspired by the chosen-plaintext attack, we propose an efficient reference-validation inference algorithm to break the random mapping sequence, and design screening-based rules to determine the keys of the orthogonal Latin cubes. In total, our cryptanalysis algorithm requires only$\;\sqrt[{3}]{8\times H\times W}+3\;$pairs of chosen plain-cipher images to break the TIC [13], where *H* and *W* represent the image’s height and width, respectively. 

### 3.1. Vulnerability Analysis

Although Xu and Tian [13] conducted various security tests, there still exist four fatal vulnerabilities in their image cryptosystem. First, the process of generating the chaotic sequence **r** is independent of the plain image, so that an attacker can arbitrarily fabricate desired plain images without influencing **r**. Second, the diffusion phase, as shown in Equation (6), fundamentally inherits from the traditional CBC mode. This means that modifying one bit in the plain image may only affect a small part of the diffused bits. In such circumstance, an attacker can mine useful information, for example the number of unchanged bits, to infer the random mapping sequence **s**. Third, the initial value of the diffusion phase is set to *v*(−1) = 0 without introducing any cryptography mechanism. This flaw somewhat eases the cryptanalysis task. Fourth, the post-permutation phase, as shown in Equation (9), fails to conceal the statistical information of **V**. This enables an attacker to bypass the post-permutation phase and to calculate the equivalent CPV directly from the cipher images.

Kerckhoffs’s principle, which lies at the core of cryptanalysis, sets forth that the security of a cryptosystem relies only on the keys, rather than on the details of the encryption/decryption algorithm. In other words, the encryption/decryption details, e.g., the coordinate transformations between 1D and 3D as shown in Equations (5) and (8), are all open knowledge. In summary, the task of breaking the TIC [13] is equivalent to reconstructing the key-based information, including the random mapping sequence **s** (which is controlled by the keys *κ* and *μ*), and the three orthogonal Latin cubes **L**_1_, **L**_2_, and **L**_3_ (which are controlled by the keys *α*, *β*, and *γ*). 

Hereafter, we act as an attacker and use the existing vulnerabilities to break the TIC [13]. In Section 3.2, we ascertain what controls the CPV and propose a reference-validation inference algorithm to reconstruct **s**. In Section 3.3, we turn to design screening-based rules to determine the keys *α*, *β*, and *γ*.

### 3.2. Reference-Validation Inference Algorithm

#### 3.2.1. Simplify the Pre-Permutation Phase

As discussed in [22], some special coordinates in the plain bit-cube **P** can be used to simplify the pre-permutation phase. When substituting *s*(*x*) = *s*(*y*) = 0 into Equation (2), we have *L*_1_(*x*, *y*, *z*) = *L*_2_(*x*, *y*, *z*) = *L*_3_(*x*, *y*, *z*) = *s*(*z*). Then, the pre-permutation phase, as shown in Equations (2) and (3), can be rewritten as

*U*(*x*, *y*, *z*) = *P*(*s*(*z*), *s*(*z*), *s*(*z*)), (10)

in which *s*(*z*) must be an integer lying in the interval [0, *N −* 1]. Here, we introduce a new notation *s*^−1^(·) to represent the inverse of *s*(·), and further formulate Equation (10), giving

*U*(*s*^−1^(0), *s*^−1^(0), *s*^−1^(*n*)) = *P*(*n*, *n*, *n*), (11)

where the coordinate (*n*, *n*, *n*) is located at the diagonal of **P**. In other words, as long as we visit the diagonal bits of **P**, the original pre-permutation phase can be simplified to Equation (11). This paves the way for inferring the mapping relations between *s*^−1^(*n*) and *n*. As doing so, reconstructing **s** is trivial since *s*^−1^(·) belongs to a bijective mapping.

To this end, the first step is to choose the plain images, which can simplify the pre-permutation phase according to Equation (11). We create *N* plain bit-cubes by modifying the diagonal bits of **P** in turn. This procedure is described as follows

(12) $$P_{n}^{\prime }(x,\;y,\;z)=\{\begin{array}{ll} 1-P(x,\;y,\;z)\; & \mathrm{if}\;x=y=z=n,\;\\ \;P(x,\;y,\;z) & \mathrm{otherwise},\; \end{array}$$

where *n* = 0, 1, ⋯, *N* − 1. The notation${\;P}_{n}^{\prime }={[P}_{n}^{\prime }\left(x,\;y,\;z\right)|x,\;y,\;z\;=\;0,\;1,\;\cdots \;,\;N-1]\;$represents the *n*th created plain bit-cube. Clearly, the only one different bit between **P** and${\;P}_{n}^{\prime }\;$is located at the coordinate (*n*, *n*, *n*). As discussed above, modifying bit values in **P** will not influence the chaotic behaviors of the Logistic map so that all these plain bit-cubes, namely **P** and${\;P}_{n}^{\prime }$, share the same **s**. This provides us with the opportunity to break the random mapping sequence **s** by the chosen-plaintext attack. For the convenience of presentation, we shall attach a prime superscript on the letter to signify the intermediate encryption result of${\;P}_{n}^{\prime }$. For example, the symbols${\;U}_{n}^{\prime }$,${\;V}_{n}^{\prime }$,${\;C}_{n}^{\prime }\;$correspond to the pre-permutated, the diffused and the post-permutated version of${\;P}_{n}^{\prime }$, respectively.

Feeding${\;P}_{n}^{\prime }\;$into the TIC [13], we can obtain the post-permutated bit-cube${\;C}_{n}^{\prime }$. The new CPV, denoted by$\;t_{n}^{\prime }$, can be directly computed from$\;C_{n}^{\prime }\;$even without knowing$\;V_{n}^{\prime }\;$because the post-permutation phase does not affect any bit values. According to whether$\;t_{n}^{\prime }=t\;$or$\;t_{n}^{\prime }\neq t$, the *N* pairs of chosen plain-cipher images can be divided into CPV-preserving and CPV-changing groups. The two kinds of groups will be tackled through different strategies, as will be presented below.

#### 3.2.2. CPV-Preserving Group

**Figure 2:**
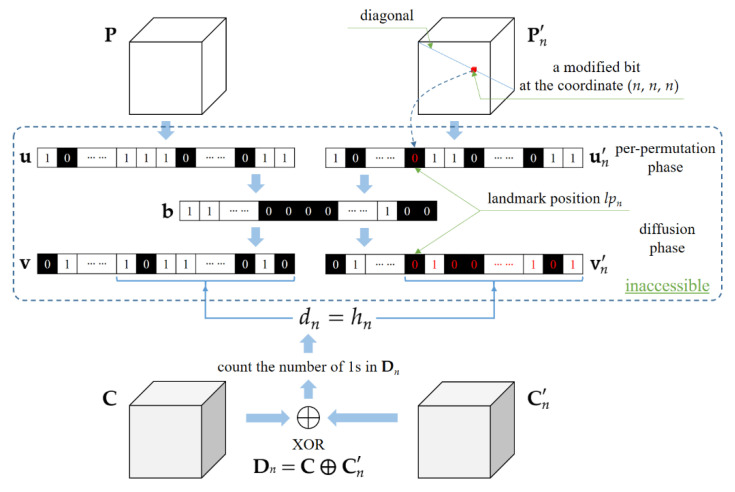
Illustration of calculating$\;d_{n}$,$\;h_{n}$ and$\;{lp}_{n}$ when$\;t_{n}^{\prime }=t$. Note that the intermediate encryption results within the dotted box, such as **u** or **v**, are inaccessible for the attacker. The flipped bit values are expressly marked by red ink.

When${\;t}_{n}^{\prime }=t$, the bit-cubes **C** and${\;C}_{n}^{\prime }\;$have undergone the same post-permutation phase, as shown in Equation (9), so that we can directly apply the bit-wise XOR operator to them. Calculate a differential bit-cube$\;D_{n}=C\;⨁{\;C}_{n}^{\prime }$, and count the total number of 1s in$\;D_{n}$. Let$\;d_{n}\;$be the counting result. Since the diffusion phase, as shown in Equation (6), belongs to the weak CBC mode, a modified bit in$\;u_{n}^{\prime }\;$will affect the diffused sequence$\;v_{n}^{\prime }\;$starting from the landmark position and propagating the influence of the modification along the way forward to the end, as illustrated in Figure 2. Here, the landmark position, denoted by$\;{lp}_{n}$, plays a dual role. On one hand, it corresponds to the flipped bit in$\;u_{n}^{\prime }\;$and to the modified bit in${\;P}_{n}^{\prime }\;$at the coordinate (*n*, *n*, *n*), as indicated by the dotted arrow in Figure 2. Hence, the landmark position$\;{lp}_{n}\;$can be represented by

(13) $${lp}_{n}={\;s}^{-1}\left(0\right)\times N^{2}+s^{-1}\left(0\right)\times N+s^{-1}\left(n\right).$$

Equation (13) is a 3D-to-1D coordinate transformation, which instantiates Equation (5) by using (*s*^−1^(0), *s*^−1^(0), *s*^−1^(*n*)) for (*x*, *y*, *z*). On the other hand, the landmark position can reflect the number of flipped bits in${\;v}_{n}^{\prime }$, denoted by$\;h_{n}$, taking the following form:

(14) $${lp}_{n}=N^{3}-h_{n},$$

where${\;N}^{3}\;$is the total number of bits. As illustrated in Figure 2, when${\;t}_{n}^{\prime }=t\;$holds, the number of flipped bits$\;h_{n}\;$can be exposed by counting the number of 1s in $D_{n}$, namely that the equation$\;h_{n}=d_{n}\;$holds. Substitute for$\;{lp}_{n}\;$in Equation (13) using Equation (14) and apply the 1D-to-3D coordinate transformation, giving

(15) $$\;\{\begin{array}{l} s^{-1}\left(0\right)=⎣{(N}^{3}-h_{0})/N^{2}⎦=⎣{(N}^{3}-h_{0})/N⎦\%N,\\ s^{-1}{(n)=(N}^{3}-h_{n})\%N.\;\; \end{array}$$

Equation (15) establishes the relationship between *s*^−1^(*n*) and$\;h_{n}$. Based on this relationship, we can readily determine the mapping relations *s*^−1^(·) due to the one-to-one correspondence between$\;h_{n}\;$and *n*.

#### 3.2.3. What Controls the CPV

It is worth ascertaining what controls the CPV and how many pairs of chosen plain-cipher images in the CPV-preserving and CPV-changing groups, respectively. The following three propositions comprehensively assert that the last term in Equation (13), namely *s*^−1^(*n*), controls the CPV.

**Proposition** **1.**  *If the number of flipped bits, namely*$\;h_{n}$*, is even, then*$\;t_{n}^{\prime }=t$*. Otherwise*$\;t_{n}^{\prime }\neq t$.

**Proof.**  Clearly, the flipped bits in$\;v_{n}^{\prime }$, as marked by red ink in Figure 2, are the sources of changing the CPV. Hence, in this proof, we only focus on the flipped part that consists of$\;h_{n}\;$bits. Suppose that$\;h_{n}=h_{n}^{(1\to 0)}+h_{n}^{(0\to 1)}$, where${\;h}_{n}^{(1\to 0)}\;$(and${\;h}_{n}^{(0\to 1)}$) denotes the number of 1s (and 0s) being flipped to 0 (and 1) caused by the one bit modification at the landmark position.

If$\;h_{n}\;$is even, we will encounter one of the following two cases: (1) both$\;h_{n}^{(1\to 0)}\;$and$\;h_{n}^{(0\to 1)}\;$are even; (2) both$\;h_{n}^{(1\to 0)}\;$and$\;h_{n}^{(0\to 1)}\;$are odd. In both cases,$\;h_{n}^{(1\to 0)}\;$and$\;h_{n}^{(0\to 1)}\;$share the same parity so that flipping$\;h_{n}$ bits will not change the CPV.

On the contrary, if$\;h_{n}\;$is odd, the two cases become: (1)$\;h_{n}^{(1\to 0)}\;$is odd while$\;h_{n}^{(0\to 1)}\;$is even; (2)$\;h_{n}^{(1\to 0)}\;$is even while$\;h_{n}^{(0\to 1)}\;$is odd. In both cases,$\;h_{n}^{(1\to 0)}\;$and$\;h_{n}^{(0\to 1)}\;$possess the opposite parity. This means that the number of 1s in the to-be-flipped part of **v** will be changed from an odd integer to an even integer for case (1), and from an even integer to an odd integer for case (2). Consequently, if$\;h_{n}\;$is odd,$\;t_{n}^{\prime }\;$is necessarily opposite to *t*. □

**Proposition** **2.**  *If the landmark position*$\;{lp}_{n}\;$*is even, then*$\;t_{n}^{\prime }=t$*. Otherwise*${\;t}_{n}^{\prime }\neq t$.

**Proof.**  Equation (14) establishes the relationship between$\;h_{n}\;$and$\;{lp}_{n}$, in which$\;N^{3}=W\times H\times 8\;$is even because it is a multiple of 8. Due to this evenness, Equation (14) forces$\;h_{n}\;$and$\;{lp}_{n}\;$to share the same parity. Consequently, the landmark position$\;{lp}_{n}$, just like$\;h_{n}$, controls the CPV. □

**Proposition** **3.**  *Ifs*^−1^(*n*) *is even, then*$\;t_{n}^{\prime }=t$*. Otherwise*${\;t}_{n}^{\prime }\neq t$.

**Proof.**  Since$\;N^{3}=W\times H\times 8$,$\;N=2\cdot {\left(W\times H\right)}^{1/3}\;$and$\;N^{2}=4\cdot {\left(W\times H\right)}^{2/3}\;$are multiples of even integers, they must be even. Similarly, the first and second terms on the right-hand side of Equation (13) must be even as well because they take *N* and *N*^2^ as multipliers. The remaining two terms, namely$\;{lp}_{n}\;$and *s*^−1^(*n*), are therefore forced to share the same parity. Consequently, *s*^−1^(*n*) exactly plays the same role as$\;{lp}_{n}\;$and$\;h_{n}\;$in controlling the CPV. □

Since *s*^−1^(·) must be an integer lying in the interval [0, *N −* 1], half CPVs$\;t_{n}^{\prime }\;$will remain unchanged when *s*^−1^(*n*) is even, while the remaining half ones are opposite to *t* when *s*^−1^(*n*) is odd. When$\;t_{n}^{\prime }=t$, we determine$\;h_{n}\;$by counting the number of 1s in${\;D}_{n}$, and then calculate *s*^−1^(*n*) by using Equation (15). When$\;t_{n}^{\prime }\neq t$, **V** and$\;V_{n}^{\prime }\;$will be permuted by different ways, as shown in Equation (9), so that it is meaningless to calculate the bit-wise XOR of **C** and${\;C}_{n}^{\prime }$. To alleviate this problem, we propose a reference-validation inference algorithm that requires only two additional pairs of chosen plain-cipher images to determine the mapping relations between *s*^−1^(*n*) and *n* for the CPV-changing group. See details in the next three subsections.

For future convenience, we define two index sets$\;N^{=}\;$and$\;N^{\neq }\;$with the same cardinality N/2. The former consists precisely of the indices *n* for which$\;t_{n}^{\prime }=t$ is true. Members of the latter are the indices *n* for which$\;t_{n}^{\prime }\neq t\;$is true. Properties of the CPV-preserving and CPV-changing groups are summarized in Table 1 for ease of comparison. 

#### 3.2.4. Pair of Reference Plain-Cipher Images

When$\;t_{n}^{\prime }\neq t$,$\;D_{n}=C\;⨁{\;C}_{n}^{\prime }\;$where$\;n\in N^{\neq }$, is no longer informative because the number of 1s in$\;D_{n}\;$does not reflect the number of flipped bits in$\;C_{n}^{\prime }$. We shall resort to another means to measure$\;h_{n}$. The reference-validation inference algorithm fulfills this need through the following three steps

[step-1]: using a pair of reference plain-cipher images to detect leftmost and rightmost landmark positions for the CPV-changing group (discussed in this subsection);
[step-2]: using a pair of validation plain-cipher images to determine the index $n\in N^{\neq }$, whose$\;{lp}_{n}\;$corresponds to the leftmost landmark position (discussed in Section 3.2.5);
[step-3]: using the leftmost landmark position to measure$\;h_{n}$, where *n*$\;\in N^{\neq }$ (discussed in Section 3.2.6).

To achieve step-1, we create the reference plain bit-cube by simultaneously modifying three diagonal bits of **P**

(16) $$P^{\mathrm{ref}}(x,\;y,\;z)=\{\begin{array}{ll} 1-P(x,\;y,\;z)\; & {\mathrm{if}\;x=y=z=n}^{\mathrm{left}},\;\\ 1-P(x,\;y,\;z)\; & {\mathrm{if}\;x=y=z=n}^{\mathrm{mid}},\;\\ 1-P(x,\;y,\;z)\; & {\mathrm{if}\;x=y=z=n}^{\mathrm{right}},\;\\ P(x,\;y,\;z) & \mathrm{otherwise},\;\; \end{array}$$

where$\;P^{\mathrm{ref}}=\left[P^{\mathrm{ref}}\left(x,\;y,\;z\right)|x,\;y,\;z=0,\;1,\;\cdots \;,\;N-1\right]\;$denotes the reference plain bit-cube. Hereafter, a letter with the “ref” superscript, such as$\;V^{\mathrm{ref}}\;$or$\;C^{\mathrm{ref}}$, signifies the intermediate encryption result of$\;P^{\mathrm{ref}}$. Most importantly, the three indices, namely$\;n^{\mathrm{left}}$,$\;n^{\mathrm{mid}}\;$and$\;n^{\mathrm{right}}$, are constrained to satisfy the following two requirements

(i) $n^{\mathrm{left}}\;$and$\;n^{\mathrm{right}}\;$are selected from$\;N^{\neq }\;$while$\;n^{\mathrm{mid}}\;$comes from$\;N^{=}$;
(ii) the corresponding three landmark positions satisfy the inequality$\;{lp}_{n^{\mathrm{left}}}<{lp}_{n^{\mathrm{mid}}}<{lp}_{n^{\mathrm{right}}}$.

**Figure 3:**
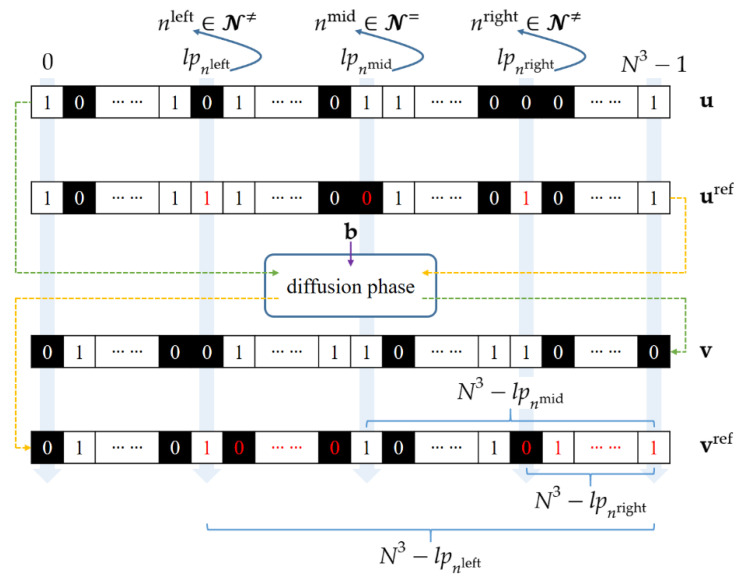
Illustration of the three modified bits in$\;u^{\mathrm{ref}}$, the flipped bits in$\;v^{\mathrm{ref}}$, and the three landmark positions. The flipped bit values are expressly marked by red ink.

The requirement (i) implies that$\;P^{\mathrm{ref}}\;$provides a bridge between the CPV-preserving and CPV-changing groups. The requirement (ii) ensures that the new CPV, denoted by$\;t^{\mathrm{ref}}$, equals *t*. The reason for this is presented below. Since$\;{lp}_{n^{\mathrm{left}}}<{lp}_{n^{\mathrm{mid}}}<{lp}_{n^{\mathrm{right}}}$, one bit modification will affect$\;v^{\mathrm{ref}}\;$starting from$\;{lp}_{n^{\mathrm{left}}}\;$and ending at$\;{lp}_{n^{\mathrm{mid}}}$. The flipped bits appear once again starting from$\;{lp}_{n^{\mathrm{right}}}\;$until the end of the last bit of$\;v^{\mathrm{ref}}$. As illustrated in Figure 3, the flipped bits (red ink) are separated into two parts in${\;v}^{\mathrm{ref}}$. The number of flipped bits in$\;v^{\mathrm{ref}}$, denoted by$\;h^{\mathrm{ref}}$, can be represented by

(17) $$h^{\mathrm{ref}}={(N}^{3}-{lp}_{n^{\mathrm{left}}})-{(N}^{3}-{lp}_{n^{\mathrm{mid}}})+{(N}^{3}-{lp}_{n^{\mathrm{right}}})=N^{3}-{lp}_{n^{\mathrm{left}}}+{lp}_{n^{\mathrm{mid}}}-{lp}_{n^{\mathrm{right}}},$$

where$\;N^{3}\;$is an even integer. Moreover, both$\;{lp}_{n^{\mathrm{left}}}\;$and$\;{lp}_{n^{\mathrm{right}}}\;$are odd because the indices $n^{\mathrm{left}}\;$and $n^{\mathrm{right}}\;$belong to$\;N^{\neq }$. Conversely, the landmark position$\;{lp}_{n^{\mathrm{mid}}}\;$is even since$\;n^{\mathrm{mid}}\in N^{=}$. As shown in (17), the expression for$\;h^{\mathrm{ref}}\;$contains two even items and two odd items, so that$\;h^{\mathrm{ref}}\;$must be even. Thus, the evenness of$\;h^{\mathrm{ref}}\;$ensures that$\;t^{\mathrm{ref}}=t\;$according to Proposition 1.

To meet the two requirements, we design the following procedures for selecting the three indices. First, define a new differential bit-cube$\;D_{m,n}^{\prime }=C_{m}^{\prime }\;⨁{\;C}_{n}^{\prime }$, where both *m* and *n* come from$\;N^{\neq }$, and we have$\;t_{m}^{\prime }=t_{n}^{\prime }\;\neq \;t$. The number of 1s in${\;D}_{m,n}^{\prime }$, denoted by$\;d_{m,n}^{\prime }$, is informative in providing the distance between$\;{lp}_{m}\;$and$\;{lp}_{n}$, giving

(18) $$d_{m,n}^{\prime }=\left|{lp}_{m}-{lp}_{n}\right|.$$

Traverse all possible combinations {*m*, *n*} and calculate$\;d_{m,n}^{\prime }$. Search the combination that gives the maximum value in (18). That is

(19) $$m^{\neq }{,n}^{\neq }=\underset{m,n}{\mathrm{argmax}}\;d_{m,n}^{\prime },\;\mathrm{for}\;m\in N^{\neq }\;\mathrm{and}\;n\in N^{\neq },$$

where we stipulate that$\;m^{\neq }<n^{\neq }$. Next, traverse all indices$\;n\in N^{=}\;$and select the one whose landmark position is the median. That is

(20) $$n^{=}=\underset{n}{\mathrm{argmed}}\;d_{n},\;\mathrm{for}\;n\in N^{=}.$$

It is worth mentioning that the traversal operations used above are computationally feasible for$\;N^{=}\;$and$\;N^{\neq }$. This is because the cardinality of$\;N^{=}\;$(and$\;N^{\neq }$) equals N/2, which is a negligible number compared with the size of key space.

Clearly,$\;n^{\mathrm{mid}}\;$is set to$\;n^{=}$. However, there exist two candidate settings, which, in this paper, are called null and alternative hypotheses, respectively. The null hypothesis is that$\;n^{\mathrm{left}}=m^{\neq }\;$and$\;n^{\mathrm{right}}=n^{\neq }$. The alternative hypothesis is that$\;n^{\mathrm{left}}=n^{\neq }\;$and$\;n^{\mathrm{right}}=m^{\neq }$. Which of these two hypotheses is true will be determined by using the pair of validation plain-cipher images, as will be discussed later. At this moment, we break the tie by taking the null hypothesis as a provisional setting. 

With these preparations, we detect the leftmost and rightmost landmark positions as follows. First, the equality$\;t^{\mathrm{ref}}=t\;$enables us to define a reference differential bit cube$\;D^{\mathrm{ref}}\;$by applying the bit-wise XOR operator to **C** and$\;C^{\mathrm{ref}}$, namely$\;D^{\mathrm{ref}}=C\;⨁{\;C}^{\mathrm{ref}}$. The number of 1s in$\;D^{\mathrm{ref}}$, denoted by$\;d^{\mathrm{ref}}$, equals$\;h^{\mathrm{ref}}$. That is

(21) $$d^{\mathrm{ref}}=h^{\mathrm{ref}}=N^{3}-{lp}_{n^{\mathrm{left}}}+{lp}_{n^{\mathrm{mid}}}-{lp}_{n^{\mathrm{right}}}$$

in which the middle landmark position$\;{lp}_{n^{\mathrm{mid}}}\;$is known, whose value has been calculated in the last subsection. Further reformulate Equation (21) as follows

(22) $${lp}_{n^{\mathrm{right}}}+{lp}_{n^{\mathrm{left}}}=N^{3}+{lp}_{n^{\mathrm{mid}}}-d^{\mathrm{ref}},$$

where all unknowns are gathered on the left-hand side while the terms on the right-hand side are all accessible. Then, the distance between$\;{lp}_{n^{\mathrm{right}}}\;$and$\;{lp}_{n^{\mathrm{left}}}\;$can be calculated according to Equation (18), giving

(23) $${lp}_{n^{\mathrm{right}}}-{lp}_{n^{\mathrm{left}}}=d_{n^{\mathrm{left}}{,n}^{\mathrm{right}}}^{\prime },$$

where the absolute value sign has been removed because$\;{lp}_{n^{\mathrm{right}}}\;$must be greater than$\;{lp}_{n^{\mathrm{left}}}$. Combining Equations (22) and (23), we can obtain the solutions for$\;{lp}_{n^{\mathrm{left}}}\;$and$\;{lp}_{n^{\mathrm{right}}}$. 

#### 3.2.5. Pair of Validation Plain-Cipher Images

In this subsection, we conduct the hypothesis testing to determine the true values of$\;n^{\mathrm{left}}\;$and $n^{\mathrm{right}}\;$from the two candidates$\;m^{\neq }\;$and$\;n^{\neq }$. The null hypothesis is that$\;n^{\mathrm{left}}=m^{\neq }\;$and$\;n^{\mathrm{right}}=n^{\neq }$, which has been considered as a provisional setting. The alternative hypothesis is that$\;n^{\mathrm{left}}=n^{\neq }\;$and$\;n^{\mathrm{right}}=m^{\neq }$. To this end, we create the validation plain bit-cube, denoted by$\;P^{\mathrm{val}}$, by simultaneously modifying three bits at the diagonal of **P**

(24) $$P^{\mathrm{val}}(x,y,z)=\{\begin{array}{ll} 1-P(x,y,z) & \mathrm{if}x=y=z={\tilde{n}}^{\mathrm{left}\;}\\ 1-P(x,y,z) & \mathrm{if}x=y=z={\tilde{n}}^{\mathrm{mid}\;}\\ 1-P(x,y,z) & \mathrm{if}x=y=z={\tilde{n}}^{\mathrm{right}\;}\\ P(x,y,z) & \mathrm{otherwise} \end{array}$$

where$\;P^{\mathrm{val}}\left(x,\;y,\;z\right)\;$is the element of$\;P^{\mathrm{val}}\;$at the coordinate (*x*, *y*, *z*). Accordingly, a letter with the “val” superscript, such as$\;V^{\mathrm{val}}\;$or$\;C^{\mathrm{val}}$, signifies the intermediate encryption result of$\;P^{\mathrm{val}}$. The indices ${\tilde{n}}^{\mathrm{left}}$ and ${\tilde{n}}^{\mathrm{mid}}$ are set as before, namely that ${\tilde{n}}^{\mathrm{left}}=m^{\neq }$ and ${\tilde{n}}^{\mathrm{mid}}=n^{=}$. However, ${\tilde{n}}^{\mathrm{right}}$ is set as follows

(25) $${\tilde{n}}^{\mathrm{right}}={\tilde{n}}^{\neq }=\underset{n}{\mathrm{argmax}}\;d_{m^{\neq },n}^{\prime },\;\mathrm{for}n\in N^{\neq }\backslash \left\{m^{\neq },n^{\neq }\right\}.$$

Recall that Equation (19) seeks the two indices$\;m^{\neq }\;$and$\;n^{\neq }\;$that maximize$\;d_{m,n}^{\prime }$. Here, Equation (25) leaves$\;m^{\neq }\;$unchanged and seeks a new index$\;{\tilde{n}}^{\neq }\;$so that the distance between$\;{lp}_{m^{\neq }}\;$and$\;{lp}_{m^{\neq }}\;$reaches the second largest value.

**Figure 4:**
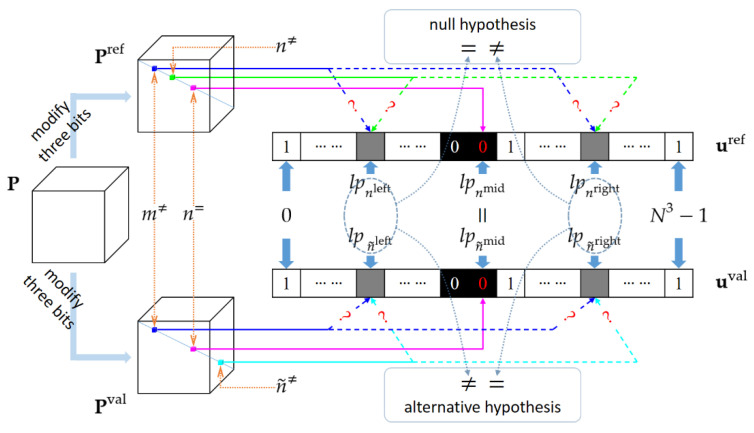
Illustration of the hypothesis testing. The solid pink arrows indicate the deterministic settings of$\;n^{=}$, while the dotted ones with question marks indicate the hypothesis settings.

Since the new indices ${\tilde{n}}^{\mathrm{left}}$, ${\tilde{n}}^{\mathrm{mid}}$ and ${\tilde{n}}^{\mathrm{right}}$ still satisfy the requirements (i) and (ii),$\;C^{\mathrm{val}}\;$and$\;C^{\mathrm{ref}}\;$are constrained to share the same CPV. We have$\;t^{\mathrm{val}}=t^{\mathrm{ref}}=t$. This allows us to define a differential bit cube$\;D^{\mathrm{val}}=C\;⨁{\;C}^{\mathrm{val}}$. Let$\;d^{\mathrm{val}}\;$denote the number of 1s in${\;D}^{\mathrm{val}}$. As done before, we can get the solutions for $lp_{{\tilde{n}}^{\mathrm{left}}}$ and $lp_{{\tilde{n}}^{\mathrm{right}}}$, where$\;d^{\mathrm{val}}\;$instead of$\;d^{\mathrm{ref}}\;$is used in Equation (22). As illustrated in Figure 4, if $lp_{{\tilde{n}}^{\mathrm{left}}}=lp_{n^{\mathrm{left}}}$ and $lp_{{\tilde{n}}^{\mathrm{right}}}\neq lp_{n^{\mathrm{right}}}$, accept the null hypothesis. Otherwise, accept the alternative hypothesis.

#### 3.2.6. CPV-Changing Group

From the CPV-changing group, the leftmost landmark position$\;{lp}_{n^{\mathrm{left}}}\;$plays a key role in determining the mapping relations between *s*^−1^(*n*) and *n*. Specifically, we state that the index$\;n^{\mathrm{left}}\;$satisfies the following equation

(26) $$n^{\mathrm{left}}=\underset{n}{\mathrm{argmin}}\;{lp}_{n},\;\mathrm{for}\;n\in N^{\neq }.$$

Equipped with this property, we can remove the absolute value sign in Equation (18), and represent the unknown landmark positions through

(27) $${lp}_{n}={lp}_{n^{\mathrm{left}}}+d_{n^{\mathrm{left}},n}^{\prime },$$

where$\;d_{n^{\mathrm{left}},n}^{\prime }\;$representing the number of 1s in$\;D_{n^{\mathrm{left}},n}^{\prime }$, has been calculated in Equation (19). Traverse each member *n*$\;\in N^{\neq }$, look up$\;d_{n^{\mathrm{left}},n}^{\prime }\;$in turn, and use Equation (27) to get$\;{lp}_{n}$. Doing so exposes all landmark positions for the parity-changing group. Further, exploit Equations (14) and (15) to determine the mapping relations between *s*^−1^(*n*) and *n*, where$\;n\in N^{\neq }$. So far, all *s*^−1^(*n*), where *n* = 0, 1, …, *N −* 1, has been obtained, enabling us to reconstruct the random mapping sequence **s** even without knowing the keys *κ* and *μ*. 

### 3.3. Screening-Based Rules

The pre-permutation phase is based on the three orthogonal Latin cubes, as shown in Equation (2), which belong essentially to quadratic equations over the ring ℤ/Nℤ. The keys, namely *α*, *β*, and *γ*, can be regarded as unknown variables from the perspective of cryptanalysis. Under the condition that **s** has been reconstructed, the three shared factors, namely *s*(*x*), *s*(*y*), and *s*(*z*), can be exposed by the chosen-plaintext attack. In other words, *s*(*x*), *s*(*y*), and *s*(*z*) are viewed as known variables of the quadratic equations in this subsection. However, there may exist multiple groups of the shared factors due to the uncertainty of the CPV. We design screening-based rules to eliminate the uncertainty, paving the way for breaking *α*, *β*, and *γ*.

To this end, we need to create two plain bit-cubes$\;P_{0}^{\prime \prime }\;$and${\;P}_{1}^{\prime \prime }\;$by modifying the bits at the coordinates (*L*_1_(*x*_0_, *y*_0_, *z*_0_), *L*_2_(*x*_0_, *y*_0_, *z*_0_), *L*_3_(*x*_0_, *y*_0_, *z*_0_)) and (*L*_1_(*x*_1_, *y*_1_, *z*_1_), *L*_2_(*x*_1_, *y*_1_, *z*_1_), *L*_3_(*x*_1_, *y*_1_, *z*_1_)), respectively. The coordinates are substituted into the left-hand side of Equation (2). To effectively solve the quadratic equations, we choose the coordinates obeying the following three conditions [24]. First, *L*_1_(*x*_0_, *y*_0_, *z*_0_) = *L*_2_(*x*_0_, *y*_0_, *z*_0_) ≠ *L*_3_(*x*_0_, *y*_0_, *z*_0_) and *L*_1_(*x*_1_, *y*_1_, *z*_1_) ≠ *L*_2_(*x*_1_, *y*_1_, *z*_1_) = *L*_3_(*x*_1_, *y*_1_, *z*_1_). Second, *L*_2_(*x*_0_, *y*_0_, *z*_0_) = *L*_2_(*x*_1_, *y*_1_, *z*_1_). Third, all these values are integers taken from the interval [0, *N −* 1].

Feed${\;P}_{m}^{\prime \prime }\;$into the TIC [13]. Obtain$\;C_{m}^{\prime \prime }\;$and$\;t_{m}^{\prime \prime }$, where m=0, 1. The number of the flipped bits in${\;C}_{m}^{\prime \prime }$, denoted by$\;h_{m}^{\prime \prime }$, is the key information to expose *s*(*x_m_*), *s*(*y_m_*) and *s*(*z_m_*). Instantiate Equation (8) by using$\;h_{m}^{\prime \prime }$, giving

(28) $$\{\begin{array}{l} s^{-1}\left(x_{m}\right)=⎣h_{m}^{\prime \prime }/N^{2}⎦,\;\\ s^{-1}\left(y_{m}\right)=⎣h_{m}^{\prime \prime }/N⎦\%N,\;\\ s^{-1}\left(z_{m}\right)=h_{m}^{\prime \prime }\%N,\; \end{array}$$

all of which can be readily converted into *s*(*x_m_*), *s*(*y_m_*) and *s*(*z_m_*) by using the knowledge of **s**. 

However, it is not trivial to obtain$\;h_{m}^{\prime \prime }$. We shall first check whether$\;t_{m}^{\prime \prime }\;$equals *t* or not. If$\;t_{m}^{\prime \prime }=t$, calculate a differential bit-cube$\;D_{m}^{\prime \prime }=C\;⨁{\;C}_{m}^{\prime \prime }$, and then count the number of 1s in${\;D}_{m}^{\prime \prime }$, denoted by$\;d_{m}^{\prime \prime }$. Similar to the CPV-preserving group, we have$\;h_{m}^{\prime \prime }=d_{m}^{\prime \prime }$. If$\;t_{m}^{\prime \prime }\neq t$, we select an index *n* from$\;N^{\neq }\;$and have$\;t_{m}^{\prime \prime }=t_{n}^{\prime }\neq t$. In this case,$\;D_{m}^{\prime \prime }\;$should be defined as$\;D_{m}^{\prime \prime }=C_{n}^{\prime }\;⨁{\;C}_{m}^{\prime \prime }$, and$\;d_{m}^{\prime \prime }\;$represents the distance between$\;{lp}_{m}\;$and$\;{lp}_{n}$. Similar to Equation (18), we have

(29) $$d_{m}^{\prime \prime }=\left|{lp}_{m}-{lp}_{n}\right|,$$

where the absolute value sign is necessary. The reason for this is presented as follows. In this subsection, the coordinates no longer lie on the diagonal of **P**, meaning that the pre-permutation phase cannot be simplified. As a result, the statement that$\;{lp}_{n^{\mathrm{left}}}\;$(${lp}_{n^{\mathrm{right}}}$) is located at the leftmost (rightmost) side is no longer true. For an arbitrarily selected$\;n\in N^{\neq }$, its landmark position$\;{lp}_{n}\;$may be located at the left or right side of$\;{lp}_{m}$.

According to Equation (14),$\;{lp}_{m}\;$and$\;{lp}_{n}\;$can be expressed as$\;N^{3}-h_{m}^{\prime \prime }\;$and$\;N^{3}-h_{n}$, respectively. Based on this expression, we can rewrite Equation (29) in the form

(30) $$h_{m}^{\prime \prime }=h_{n}\pm d_{m}^{\prime \prime },$$

where both$\;h_{n}\;$and$\;d_{m}^{\prime \prime }\;$are accessible. Equation (30) means that, when$\;t_{m}^{\prime \prime }\neq t$, the calculation of$\;h_{m}^{\prime \prime }$ involves uncertainty, resulting in two possible values. Accordingly, by using Equation (28) and the knowledge of **s**, we may obtain two groups of the shared factors, denoted by {*s*^+^(*x_m_*), *s*^+^(*y_m_*), *s*^+^(*z_m_*)} and {*s*^−^(*x_m_*), *s*^−^(*y_m_*), *s*^−^(*z_m_*)}, respectively. Here, the superscripts are intended to signify which of the two operators “+” and “−” is used during the calculation of$\;h_{m}^{\prime \prime }$. Since it is difficult to forecast the CPV at the stage of creating$\;P_{0}^{\prime \prime }\;$and$\;P_{1}^{\prime \prime }$, we would have to consider following three cases, and design screening-based rules separately to determine the real solutions of *α*, *β*, and *γ*.

**Figure 5:**
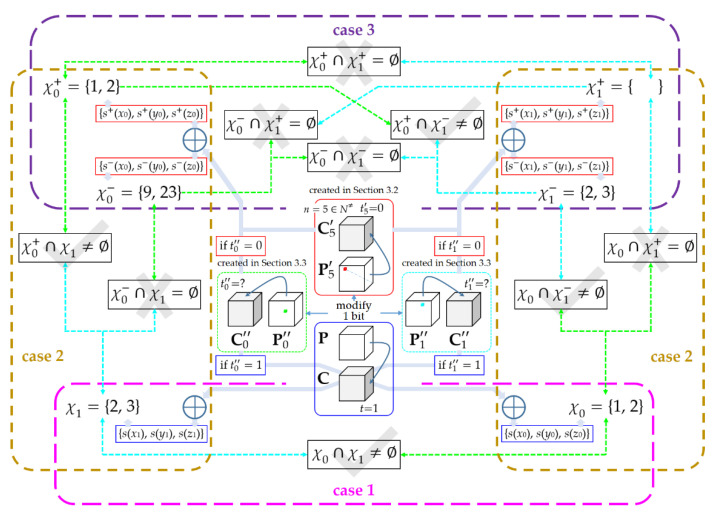
A toy example that illustrates the screening-based rules for the three cases. Suppose that the keys *α*, *β*, and *γ* take values 1, 2, and 3, respectively. The bold dotted boxes in different colors highlight the scope of each case. The gray tick and cross indicate that the group of solutions will be accepted and discarded, respectively.

The first case, indicated by “case 1” in Figure 5, occurs when$\;t_{0}^{\prime \prime }=t\;$and$\;t_{1}^{\prime \prime }=t$. In this case, we obtain that$\;h_{0}^{\prime \prime }=d_{0}^{\prime \prime }\;$and$\;h_{1}^{\prime \prime }=d_{1}^{\prime \prime }\;$without uncertainty, meaning that the shared factors {*s*(*x*_0_), *s*(*y*_0_), *s*(*z*_0_)} and {*s*(*x*_1_), *s*(*y*_1_), *s*(*z*_1_)} are both authentic. Therefore, the quadratic equations can be written as

(31) $$\{\;\begin{array}{l} L_{1}(x_{0},\;y_{0},\;z_{0})=L_{2}(x_{0},\;y_{0},\;z_{0})=\chi_{0}^{2}{\times s(x_{0})+\chi }_{0}\times \;s(y_{0})+s(z_{0})\\ L_{2}(x_{1},\;y_{1},\;z_{1})=L_{3}(x_{1},\;y_{1},\;z_{1})=\chi_{1}^{2}{\times s(x_{1})+\chi }_{1}\times \;s(y_{1})+s(z_{1}) \end{array}$$

where$\;\chi_{0}\;$and$\;\chi_{1}\;$are used here to represent the unknown variables in Equation (2). Solve Equation (31) over the ring ℤ/Nℤ and obtain$\;\chi_{0}=\left\{\chi_{0}{(0),\;\chi }_{0}\left(1\right)\right\}\;$and$\;\chi_{1}=\left\{\chi_{1}{(0),\;\chi }_{1}\left(1\right)\right\}$, each of which must contain two real solutions due to the authenticity of the shared factors [24]. For this case, the screening-based rule is to assign$\;\beta =\chi_{0}\cap \chi_{1}$,$\;\alpha =\chi_{0}\backslash \{\beta \}$, and$\;\gamma =\chi_{1}\backslash \{\beta \}$, which directly breaks the keys.

The second case, indicated by “case 2” in Figure 5, occurs when$\;t_{0}^{\prime \prime }=t\;$but$\;t_{1}^{\prime \prime }\neq t\;$(or equivalently$\;t_{0}^{\prime \prime }\neq t\;$but$\;t_{1}^{\prime \prime }=t$). In this case, {*s*(*x*_0_), *s*(*y*_0_), *s*(*z*_0_)} is still authentic but we will obtain {*s*^+^(*x*_1_), *s*^+^(*y*_1_), *s*^+^(*z*_1_)} and {*s*^−^(*x*_1_), *s*^−^(*y*_1_), *s*^−^(*z*_1_)} due to the uncertainty of$\;h_{1}^{\prime \prime }$. Hence, the quadratic equations take two possible forms

(32) $$\{\;\begin{array}{l} L_{1}(x_{0},\;y_{0},\;z_{0})\;=\;L_{2}(x_{0},\;y_{0},\;z_{0})\;=\;\chi_{0}^{2}{\;\times \;s(x_{0})\;+\;\chi }_{0}\times \;s(y_{0})\;+\;s(z_{0})\\ L_{2}\left(x_{1},y_{1},z_{1}\right)=L_{3}\left(x_{1},y_{1},z_{1}\right)={\left(\chi_{1}^{+}\right)}^{2}\times s^{+}\left(x_{1}\right)+\chi_{1}^{+}\times s^{+}\left(y_{1}\right)+s^{+}\left(z_{1}\right) \end{array}$$

(33) $$\{\;\begin{array}{l} L_{1}(x_{0},\;y_{0},\;z_{0})\;=\;L_{2}(x_{0},\;y_{0},\;z_{0})=\chi_{0}^{2}{\;\times \;s(x_{0})\;+\;\chi }_{0}\times \;s(y_{0})\;+\;s(z_{0})\\ L_{2}\left(x_{1},\;y_{1},\;z_{1}\right)=L_{3}\left(x_{1},\;y_{1},\;z_{1}\right)={\left(\chi_{1}^{-}\right)}^{2}\times s^{-}\left(x_{1}\right)+\chi_{1}^{-}\times s^{-}\left(y_{1}\right)+s^{-}\left(z_{1}\right) \end{array}$$

Solving (32) yields$\;\chi_{0}\;$and$\;\chi_{1}^{+}$. Accordingly,$\;\chi_{0}\;$and$\;\chi_{1}^{-}\;$are the solutions of Equation (33). Due to its authenticity,$\;\chi_{0}\;$can be used to screen out the real solution$\;\chi_{1}\;$from$\;\chi_{1}^{+}\;$and$\;\chi_{1}^{-}$. Specifically, the screening-based rule is that, if$\;\chi_{0}\cap \chi_{1}^{+}=\emptyset$, then$\;\chi_{1}=\chi_{1}^{-}$, otherwise$\;\chi_{1}=\chi_{1}^{+}$. In this way, the uncertainty can be eliminated, allowing us to smoothly break the keys as described before.

The third case, indicated by “case 3” in Figure 5, occurs when$\;t_{0}^{\prime \prime }\neq t\;$and$\;t_{1}^{\prime \prime }\neq t$. In this case, both$\;h_{0}^{\prime \prime }\;$and$\;h_{1}^{\prime \prime }\;$have two possible values, each of which generates two groups of the shared factors. Hence, the quadratic equations take one of the following four forms

(34) $$\{\;\begin{array}{l} L_{1}(x_{0},\;y_{0},\;z_{0})=L_{2}(x_{0},\;y_{0},\;z_{0})={\left(\chi_{0}^{+}\right)}^{2}\times s^{+}\left(x_{0}\right)+\chi_{0}^{+}\times s^{+}\left(y_{0}\right)+s^{+}\left(z_{0}\right)\\ L_{2}\left(x_{1},\;y_{1},\;z_{1}\right)=L_{3}\left(x_{1},\;y_{1},\;z_{1}\right)={\left(\chi_{1}^{+}\right)}^{2}\times s^{+}\left(x_{1}\right)+\chi_{1}^{+}\times s^{+}\left(y_{1}\right)+s^{+}\left(z_{1}\right) \end{array}$$

(35) $$\{\;\begin{array}{l} L_{1}(x_{0},\;y_{0},\;z_{0})=L_{2}(x_{0},\;y_{0},\;z_{0})={\left(\chi_{0}^{+}\right)}^{2}\times s^{+}\left(x_{0}\right)+\chi_{0}^{+}\times s^{+}\left(y_{0}\right)+s^{+}\left(z_{0}\right)\\ L_{2}\left(x_{1},\;y_{1},\;z_{1}\right)=L_{3}\left(x_{1},\;y_{1},\;z_{1}\right)={\left(\chi_{1}^{-}\right)}^{2}\times s^{-}\left(x_{1}\right)+\chi_{1}^{-}\times s^{-}\left(y_{1}\right)+s^{-}\left(z_{1}\right) \end{array}$$

(36) $$\{\;\begin{array}{l} L_{1}(x_{0},\;y_{0},\;z_{0})=L_{2}(x_{0},\;y_{0},\;z_{0})={\left(\chi_{0}^{-}\right)}^{2}\times s^{-}\left(x_{0}\right)+\chi_{0}^{-}\times s^{-}\left(y_{0}\right)+s^{-}\left(z_{0}\right)\\ L_{2}\left(x_{1},\;y_{1},\;z_{1}\right)=L_{3}\left(x_{1},\;y_{1},\;z_{1}\right)={\left(\chi_{1}^{+}\right)}^{2}\times s^{+}\left(x_{1}\right)+\chi_{1}^{+}\times s^{+}\left(y_{1}\right)+s^{+}\left(z_{1}\right) \end{array}$$

(37) $$\{\;\begin{array}{l} L_{1}(x_{0},\;y_{0},\;z_{0})=L_{2}(x_{0},\;y_{0},\;z_{0})={\left(\chi_{0}^{-}\right)}^{2}\times s^{-}\left(x_{0}\right)+\chi_{0}^{-}\times s^{-}\left(y_{0}\right)+s^{-}\left(z_{0}\right)\\ L_{2}\left(x_{1},\;y_{1},\;z_{1}\right)=L_{3}\left(x_{1},\;y_{1},\;z_{1}\right)={\left(\chi_{1}^{-}\right)}^{2}\times s^{-}\left(x_{1}\right)+\chi_{1}^{-}\times s^{-}\left(y_{1}\right)+s^{-}\left(z_{1}\right) \end{array}$$

from which we explicitly obtain four groups of solutions, denoted by {$\chi_{0}^{+}$,$\;\chi_{1}^{+}$}, {$\chi_{0}^{+}$,$\;\chi_{1}^{-}$}, {$\chi_{0}^{-}$,$\;\chi_{1}^{+}$}, and {$\chi_{0}^{-}$,$\;\chi_{1}^{-}$}, respectively. Inspect each group, the screening-based rule makes the following judgement. If the intersection of the two solutions is empty, then the corresponding group will be discarded, otherwise, it must be the authentic one {$\chi_{0}$,$\;\chi_{1}$}. For example, if only the second group has a non-empty intersection, namely that$\;\chi_{0}^{+}\cap \chi_{1}^{-}\neq \emptyset$, then we set$\;\chi_{0}=\chi_{0}^{+}\;$and$\;\chi_{1}=\chi_{1}^{-}$. By doing so, the double uncertainties can be eliminated as well. 

Figure 5 shows a toy example that illustrates the screening-based rules for the three cases. Assume that *n* = 5 is selected from$\;N^{\neq }$, and the pair of chosen plain-cipher bit-cubes, namely$\;P_{5}^{\prime }\;$and $C_{5}^{\prime }$, plays a role when$\;t_{m}^{\prime \prime }\neq t$. Note that, since the shared factors may be incorrect, there exists empty solution, such as$\;\chi_{1}^{+}\;$as shown in this example. The empty solution can be discarded directly. 

### 3.4. Performance Analysis

In total, the proposed cryptanalysis algorithm requires$\;2\times \sqrt[{3}]{H\times W}+3\;$pairs of chosen plain-cipher images to break the TIC [13]. Compared with Zhang’s work [22] that requires$\;2.5\times \sqrt[{3}]{H\times W}+6$ pairs, our method is more efficient especially when the image has a high resolution. This merit is particularly useful when the number of admissible accesses to a TIC is limited.

To reconstruct **s**, the proposed reference-validation inference algorithm requires$\;2\times \sqrt[{3}]{H\times W}+1$ pairs of chosen plain-cipher bit-cubes. First, we need to create$\;2\times \sqrt[{3}]{H\times W}-1\;$plain bit-cubes$\;P_{n}^{\prime }\;$using Equation (12), where n=1, 2, ⋯, N−1. In practice, the plain bit-cube${\;P}_{0}^{\prime }\;$is needless because$\;s^{-1}\left(0\right)$ can be immediately derived from Equation (15) regardless of the value of *n*. Second, to tackle the CPV-changing group, we create a reference plain bit-cube${\;P}^{\mathrm{ref}}\;$using Equation (16) and a validation counterpart$\;P^{\mathrm{val}}\;$using Equation (24).

To break the keys *α*, *β*, and *γ*, Zhang’s work [22] requires two, four or six pairs of chosen plain-cipher images, respectively, to deal with the three cases described in the last section. Note that when evaluating the performance of a cryptanalysis algorithm, we always consider the worst bound that need the most computational consumptions. From this perspective, we state that six pairs are needed in Zhang’s work [22]. By contrast, the designed screening-based rules can eliminate the uncertainty in$\;h_{m}^{\prime \prime }$ by fully mining the exclusion and intersection relationships between the group-wise solutions. Therefore, in our work, only two pairs of chosen plain-cipher images are sufficient to break the keys *α*, *β*, and *γ*, whichever case we encounter in practice. 

Furthermore, the number of necessary pairs of chosen plain-cipher images can be treated as a constant with respect to the keys, the positions of chosen bits or the contents of plain images. This merit allows an attacker to accurately estimate the computational consumptions before launching the attacks. The experimental results in Section 4.3 will corroborate the above claims. 

## 4. Experimental Results

In this section, we conduct simulation experiments and comparative studies to demonstrate the effectiveness and the superiority of the proposed cryptanalysis algorithm. The first experiment aims to exhibit the cryptanalysis results for five standard grayscale images. The second experiment tests the cryptanalysis performance for a camera-based natural scene image, showing the prospects for practical applications. The third experiment is devoted to the comparative studies, which demonstrates that our cryptanalysis algorithm requires fewer pairs of chosen plain-cipher images than Zhang’s work [22]. Following the setting in [13], we specify that the default keys are κ=0.12345678912341, μ=3.99999, α=1, β=2, and γ=3, respectively. Unless explicitly stated, the TIC [13] is governed by the default keys. All experiments are implemented on a desktop computer with a 2.90 GHz Intel i7-10700 central processing unit, 16.00 GB memory. The programming environment for simulations is Matlab R2017a installed on the Window 10 operation system.

### 4.1. Results of the Cryptanalysis Algorithm

**Figure 6:**
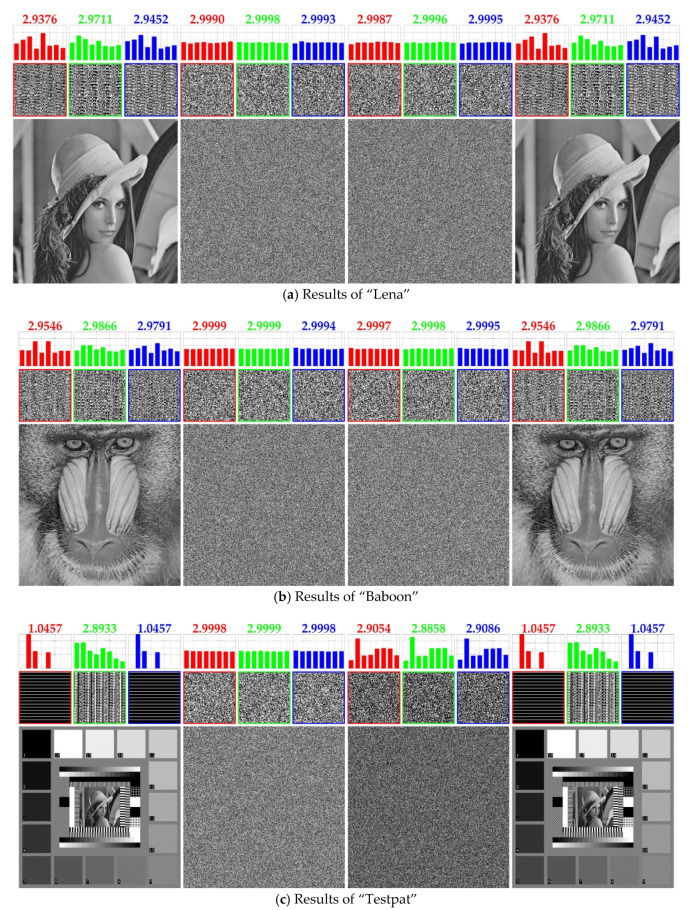

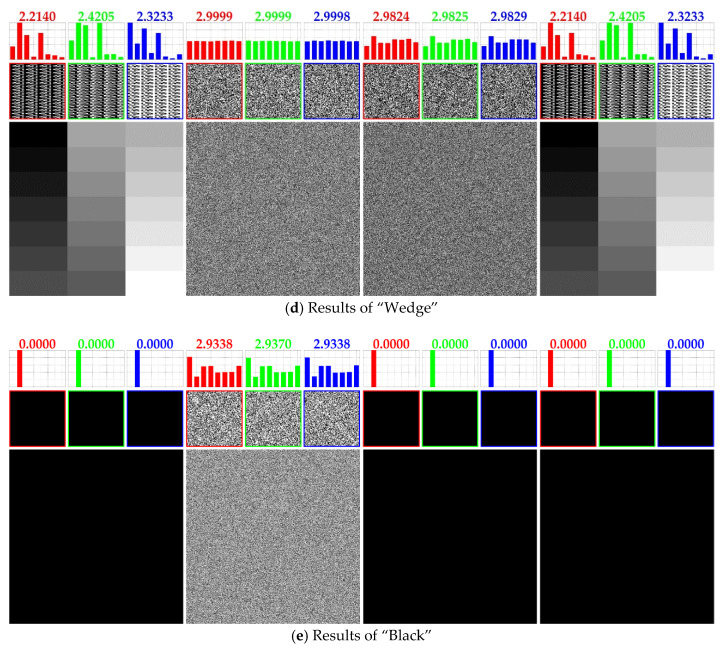
Simulation results for the first experiment. From left to right, the four columns display plain images, cipher images, partially decrypted images, and completely decrypted images, respectively. Some auxiliary information is shown in the upper panel of each image. See the texts for details.

Figure 6 shows the results for the first experiment. The first and second columns display the plain images and the corresponding cipher images obtained from the TIC [13], respectively. Five plain images are “Lena”, “Baboon”, “Testpat”, “Wedge”, and “Black”, all of which have the size of 512 × 512 × 1 (grayscale) and the side length *N* being equal to$\;\sqrt[{3}]{512\;\times \;512\;\times \;8}=128$. In this experiment, we first perform the proposed cryptanalysis algorithm for reconstructing **s** and breaking the keys *α*, *β*, and *γ*. Then, for each cipher image, perform the decryption algorithm of [13] governed by the broken information. In the third column of Figure 6, we provide the intermediate results, in which the cipher images are partially decrypted to counteract the post-permutation and diffusion phases (with the exception of the pre-permutation phase). The rightmost column shows the completely decrypted images. We see that these completely decrypted images are exactly the same as the plain images in the first column without any visual loss. For “Black”, however, the partial decryption can entirely reveal the visual information because “Black” is immune to the pre-permutation phase. As expected, the third column of Figure 6e is an all zero-valued image, which is the same as the plain image. This provides us with a new perspective to verify the correctness of our cryptanalysis algorithm on the basis of the intermediate results.

In the upper panel of each image, we append some auxiliary information, intended to provide qualitative and quantitative indicators for monitoring the progress and validating the correctness of our cryptanalysis algorithm. The auxiliary information includes CSBPs (cross-sectional bit-planes), LBP (local binary pattern) histograms, and corresponding entropy values calculated from the LBP histograms.

For qualitative comparisons, we select the front CSBP *P*(0, :, :), the middle CSBP *P*(N/2−1, :, :), and the back CSBP *P*(N−1, :, :) out of a given bit-cube **P**, where the colon operator returns a regularly-spaced vector [0, 1, …, N−1]. Intuitively, the CSBP can reflect the bit correlation in a given bit-cube. Observing the CSBPs of the plain images, we see that there exist regular LBPs, especially for “Testpat” and “Wedge”. In contrast, the cipher images’ CSBPs consist of pseudorandom LBPs, meaning that the regularity has been eliminated by the TIC [13]. Most importantly, by comparative observations, we find that the same regular LBPs reappear for the completely decrypted images, thereby verifying that the proposed cryptanalysis algorithm is capable of breaking the TIC [13].

**Figure 7:**
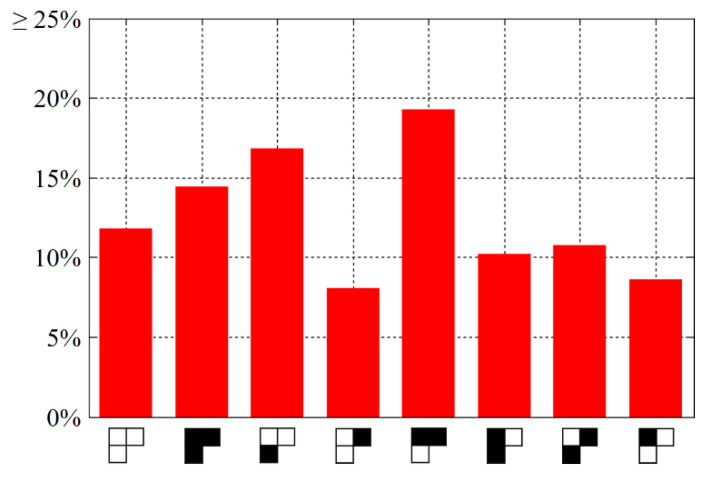
An example of the LBP (local binary pattern) histogram. This is an enlarged version derived from the upper panel of the plain image “Lena”.

In order to better characterize the regularity, we define a histogram that reflects the probability distribution of occurrence of eight LBPs in a CSBP. As shown in Figure 7, the horizontal axis of the histogram lists the eight LBPs, where three adjacent bits with different binary combinations are viewed as patterns. The vertical axis corresponds to the probability values. For clarity, we omit the captions of the axes when displaying the LBP histograms in the upper panel. Furthermore, to quantificationally measure the regularity, we calculate the entropy value from each LBP histogram. By comparison, we find that cipher images possess flatter LBP distributions and greater entropy values. This supports the statement that the LBPs for the cipher images have lower regularity. The LBP histograms in the fourth column of Figure 6 share the same shapes as those in the first column. Also, we obtain equal entropy values. These results demonstrate that the TIC [13] has been successfully broken by the proposed cryptanalysis algorithm.

Moreover, we conduct correlation analysis. The correlation coefficient can be viewed as a numerical indicator reflecting the consistency between the plain image and the completely decrypted image. In this experiment, we randomly select 10,000 pairs of adjacent pixels in horizontal, vertical, and diagonal directions from each image, and calculate the correlation coefficient$\;r_{pq}\;$as follows

(38) $$r_{pq}=\frac{\sum_{l=1}^{10^{4}}{(p}_{l}-E\left(p)\right)\times {(q}_{l}-E\left(q)\right)}{\sqrt{\sum_{l=1}^{10^{4}}{{(p}_{l}-E\left(p)\right)}^{2}}\sqrt{\sum_{l=1}^{10^{4}}{{(q}_{l}-E\left(q)\right)}^{2}}},$$

where$\;p_{l}\;$and$\;q_{l}\;$consists of the *l*th pair of adjacent pixels, and E(p) stands for the expectation of$\;p={\{p}_{1},\;p_{2},\cdots ,\;p_{10000}\}$. The correlation coefficient$\;r_{pq}\;$lies in the interval [−1, 1], and both 1 and −1 indicate the highest correlation while 0 no correlation. Particularly, we stipulate that$\;r_{pq}=\mathrm{NaN}\;$when$\;p_{l}=q_{l}=c$, where *c* is a constant, holds for all values of *l*. Table 2 lists the quantitative results. We find that the plain image and the completely decrypted image share the same correlation coefficients. For “Black”, the correlation coefficient of the partially decrypted image equals NaN because counteracting the post-permutation and diffusion phases is sufficient to recover the zero-valued pixels, giving$\;p_{l}=q_{l}\;=0$. These results also demonstrate the correctness of the proposed cryptanalysis algorithm.

### 4.2. Efficiency of the Cryptanalysis Algorithm

**Figure 8:**
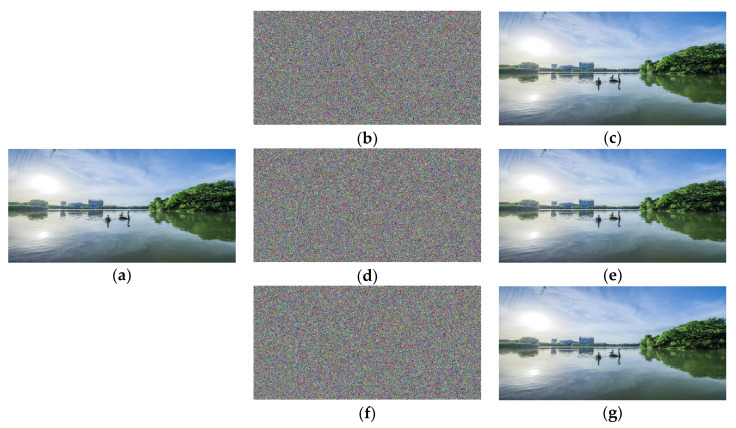
Cryptanalysis results of a camera-based natural scene image under different settings: (**a**) plain image with the size of 1024 × 2048; (**b**,**c**) results of the first setting; (**d**,**e**) results of the second setting; (**f**,**g**) results of the third setting.

In this experiment, the goal is to demonstrate the effectiveness of our cryptanalysis algorithm under a real-life application scenario. To this end, we take a landscape photograph of our university campus as the plain image. It has three color channels with the spatial resolution of 1024 × 2048. Under different settings, the TIC [13] yields three cipher images, as shown in Figure 8b,d,f, respectively. In the first setting, the three channels of the plain image are separately encrypted with the same default keys. In the second setting, we introduce a tiny change into the default keys, and separately encrypt the three channels. In the third setting, the TIC [13] is governed by three different keys, and encrypts the three channels in turn. In our computing environment, the proposed cryptanalysis algorithm takes 86.43 s, 87.15 s and 267.58 s (about 89.19 s for each channel, on average), respectively, to complete the cryptanalysis task under the three settings. Figure 8c,e,g exhibit the cryptanalysis results, in which the decrypted images are the same as the plain image.

We summarize following two points from the above experimental results. First, the proposed cryptanalysis algorithm is of high efficiency for the camera-based natural scene image, showing the feasibility of deployment in some practical system. Second, the efficiency of the proposed cryptanalysis algorithm is relatively stable. The time-consuming data tell us that different keys have almost no effect on the efficiency.

### 4.3. Comparative Studies

Zhang’s work [22] also focuses on attacking the same TIC [13]. Compared with this work [22], the proposed cryptanalysis algorithm requires fewer pairs of chosen plain-cipher images. We conduct two comparative studies as follows.

In the first comparative study, we count how many pairs of chosen plain-cipher images are necessary to break the TIC [13]. Plain images are the same as those used in Figure 6. The experimental protocol consists of the following steps. Randomly set two coordinates (*L*_1_(*x_k_*, *y_k_*, *z_k_*), *L*_2_(*x_k_*, *y_k_*, *z_k_*), *L*_3_(*x_k_*, *y_k_*, *z_k_*)), where k=0, 1, according to the conditions mentioned in Section 3.3. Perform Zhang’s cryptanalysis algorithm [22] and the proposed one, respectively. Record the number of necessary pairs of chosen plain-cipher images. For each plain image, we repeat the above steps ten times, and finally calculate the average number of necessary pairs. Moreover, we consider the default keys and a new set of keys, intended to examine whether the performance of the cryptanalysis algorithms is sensitive to keys or not. The new keys are κ=0.12345678912343, μ=3.99998, α=2, β=1, and γ=4. Numerical results are listed in Table 3.

For the plain images with the size of 512 × 512, the proposed cryptanalysis algorithm only requires 131 pairs of chosen plain-cipher images. By contrast, on average, 163.88 pairs are necessary for Zhang’s work [22]. Furthermore, as we see in Table 3, the numerical results in the columns titled by “Ours” are all the same. This demonstrates that different keys, positions of chosen bits and contents of plain images will not affect the performance of our cryptanalysis algorithm. Thus, only given the images’ resolutions, our cryptanalysis algorithm allows an attacker to pre-estimate the computational consumptions more accurately before launching the attacks. 

**Figure 9:**
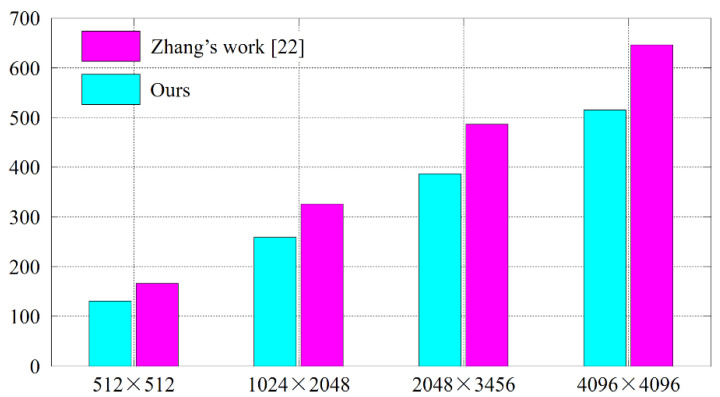
The second comparative study’s results. Lower height of a bar indicates “better”.

In the second comparative study, we aim to verify that the proposed cryptanalysis algorithm performs much more efficient when dealing with larger images. The experimental protocol consists of the following steps. Resize the plain images to 1024 × 2048, 2048 × 3456, and 4096 × 4096, respectively, using bicubic interpolation. Accordingly, the enlarged plain bit-cubes have the side length N=256, 384, and 512, respectively. For each cipher image, perform Zhang’s cryptanalysis algorithm [22] and the proposed one, respectively. Record the number of necessary pairs of chosen plain-cipher images. For the five plain images in Figure 6, we calculate the average number of necessary pairs. For comparison, the numerical results are plotted in a bar chart, as shown in Figure 9. Regardless of the resolutions, the proposed cryptanalysis algorithm consistently outperforms Zhang’s work [22], and the superiority becomes more obvious for larger images.

## 5. Conclusions

In this paper, we investigate Xu’s image cryptosystem [13], and summarize security loopholes from four aspects. On this basis, we propose a reference-validation inference algorithm and design screening-based rules to efficiently break Xu’s image cryptosystem [13]. Compared with an existing work [22], our cryptanalysis algorithm requires fewer pairs of chosen plain-cipher images. Only $\sqrt[{3}]{8\times H\times W}+3\;$pairs, where H × W represents the image’s resolution, are sufficient to complete the cryptanalysis task. Moreover, the performance of the proposed cryptanalysis algorithm is highly stable since different keys, positions of chosen bits and contents of the plain images will not influence the number of necessary pairs. This merit enables an attacker to well pre-estimate and allocate the computational consumptions before launching the attacks.

## Figures and Tables

**Table 1:** Properties of the CPV (cipher-parity value)-preserving and CPV-changing groups.

	CPV	$h_{n}$	${lp}_{n}$	*s*^−1^(*n*)	Size
CPV-preserving group	$t_{n}^{\prime }=t$	even	even	even	$\left|N^{=}\right|=N/2$
CPV-changing group	$t_{n}^{\prime }\neq t$	odd	odd	odd	$\left|N^{\neq }\right|=N/2$

**Table 2:** The numerical results for the correlation analysis.

IDs	Directions	Plain Image	Cipher Image	Decrypted Image	
				Partially	Completely
**Lena**	horizontal	0.9740	−0.0004	−0.0057	0.9740
	vertical	0.9863	−0.0025	0.0069	0.9863
	diagonal	0.9612	−0.0041	−0.0030	0.9612
**Baboon**	horizontal	0.9334	0.0027	0.0182	0.9334
	vertical	0.9102	0.0145	−0.0116	0.9102
	diagonal	0.8635	0.0011	−0.0114	0.8635
**Testpat**	horizontal	0.7395	0.0060	−0.0152	0.7395
	vertical	0.7654	−0.0107	0.0127	0.7654
	diagonal	0.7320	0.0200	−0.0200	0.7320
**Wedge**	horizontal	0.9973	0.0003	−0.0144	0.9973
	vertical	0.9998	−0.0107	−0.0229	0.9998
	diagonal	0.9971	−0.0018	0.0051	0.9971
**Black**	horizontal	NaN	0.0030	NaN	NaN
	vertical	NaN	−0.0022	NaN	NaN
	diagonal	NaN	0.0039	NaN	NaN

**Table 3:** The numerical results for the first comparative study. The better results are highlighted by underlines.

IDs	Using Default Keys		Using New Keys	
	Zhang’s Work [22]	Ours	Zhang’s Work [22]	Ours
Lena	164.0	131.0	164.0	131.0
Baboon	163.2	131.0	163.4	131.0
Testpat	163.6	131.0	163.6	131.0
Wedge	164.8	131.0	164.6	131.0
Black	163.6	131.0	164.0	131.0
